# Risk of ARI among Non-exclusively Breastfed Under-Five Passive Smoker Children: A Hospital-Based Cross-sectional Study of Nepal

**DOI:** 10.3389/fpubh.2016.00023

**Published:** 2016-02-23

**Authors:** Pushpa Thapa, Achyut Raj Pandey, Raja Ram Dhungana, Bihungum Bista, Barsha Thapa, Shiva Raj Mishra

**Affiliations:** ^1^Research Section, Nepal Family Development Foundation, Kathmandu, Nepal; ^2^Community Access Unit, Ipas Nepal, Kupondole, Nepal; ^3^Nepal Development Society, Bharatpur, Nepal

**Keywords:** acute respiratory infection, cross-sectional study, exclusive breastfeeding, household passive smoking, Nepal, under-five children

## Abstract

**Background:**

As Nepal witnesses high burden of both acute respiratory infection (ARI) and passive smoking among under-five children, studies on effect modification of exclusive breastfeeding on passive smoking and ARI carry huge significance. With Nepal holding no evidence in this regard, findings would be useful to promote a cost-effective intervention: exclusive breastfeeding. This study was therefore conceived as an effort to bring to light the modifying effect that exclusive breastfeeding may have on the association between household passive smoking and ARI among under-five children.

**Methods:**

One hundred and ninety-eight parents of under-five children from Kanti Children’s Hospital, Kathmandu, Nepal, participated in this descriptive, cross-sectional study carried out in October 2012. Data collected from a semi-structured questionnaire were subjected to univariate, bivariate, and multivariable analysis in R version 3.1.2.

**Results:**

Non-exclusively breastfed children with presence of anyone smoking at their house [aOR = 4.8, 95% confidence interval (CI): 1.7–13.2] and smoking in presence of children (aOR = 6.4, 95% CI: 1.1–38.3) had higher chances of ARI; nevertheless, this remained insignificant among the exclusively breastfed ones. Having a separate kitchen in the house showed protective effect against ARI among exclusively breastfed children (aOR = 0.2, 95% CI: 0.1–0.6). Children whose mothers spent at least 2 h per day in the kitchen had a higher chance of developing ARI, regardless of being exclusively breastfed (aOR = 4.5, 95% CI: 1.5–13.1) or non-exclusively breastfed (aOR = 4.5, 95% CI: 1.4–14.2) compared to those who spent <2 h per day.

**Conclusion:**

Non-exclusive breastfeeding may increase the chances of deleterious effects of household passive smoking, such as ARI, among under-five children. As these findings are not conclusive, studies with better design and larger samples are warranted to confirm the effect.

## Introduction

Globally, 8.8 million children under-five die each year (1), with acute respiratory infection (ARI) standing out as one of the leading causes of mortality. The year 2000 saw a death toll of two million children attributed to ARI, of which 70% were from Southeast Asia and Africa (2).

Acute respiratory infection is responsible for about 15% of deaths among children under-five in Nepal (3). Despite efforts by the Government, Nepal still has a sizeable proportion of ARI incidence among children under-five. In 2013, a total of 2,671,922 cases of ARI had been reported in Nepal, with an incidence of 951 ARI cases per 1000 under-fives (4).

Studies have established that the risk of respiratory infections increases when children are exposed to second-hand smoke (5–9). It is also important to consider that a quarter of ARI deaths in children are attributable to passive smoking (10). However, the effect of household passive smoking on ARI may differ depending on whether the baby is exclusively breastfeed or not.

Multiple studies have shown that breastfeeding enhances immunity (11–14). Human breast milk serves as a protective agent against respiratory diseases (15), and exclusive breastfeeding has been documented to lessen the chances of ARI (16, 17), especially for infants. It is possible that exclusive breastfeeding has an effect on children exposed to passive smoking. The modifying effect of exclusive breastfeeding on the apparent association between passive smoking and ARI and other respiratory symptoms among under-five children has been documented in many studies in Western countries (18–21). However, in Nepal, there is a dearth of such literature. The present study, which assessed the effect of exclusive breastfeeding on the association between household passive smoking and ARI in children under-five, was designed to address this gap.

Obtaining evidence on the modifying effect of exclusive breastfeeding on the association between household passive smoking and ARI could be advantageous in guiding policymakers to design interventions to control the overall burden of ARI in Nepal. As the country does not have universal exclusive breastfeeding practice (22), there is scope of intervention to control incidence of ARI through promotion of exclusive breastfeeding, in case, it is found to weaken the association of household passive smoking and ARI. In this context, this study assessed the effect modification of exclusive breastfeeding on association between household passive smoking and ARI.

## Materials and Methods

This was a subset analysis of the previously published hospital-based study (23). The original study examined the association between household passive smoking and ARI among under-five children.

### Study Design, Setting, and Population

This descriptive, cross-sectional study was undertaken in Kanti Children’s Hospital, Kathmandu. This particular hospital was chosen to assure the inclusion of samples from various locations across the country. Kanti Children’s Hospital is the only ­government-run tertiary level children’s hospital in Nepal. Situated in Kathmandu, the capital city of Nepal, the hospital has a capacity of 350 beds (24).

Data were collected from October 1 to 10, 2012, considering only under-five children. The parents were study respondents and were interviewed on behalf of their child.

### Sample Size and Sampling Technique

Sample size was calculated through Stat Calculator of Epi Info 7. For calculation of sample size, two-sided confidence level = 95%, power = 80%, odds ratio (OR) = 2.74 (25), ratio of children unexposed to exposed to passive smoking = 1.77 (26), percent of ARI in children not exposed to passive smoking = 30 (27), percent of ARI in children exposed to passive smoking = 50 (27), non-response rate = 10%, and control for confounding factor = 15% were considered. Sample size thus computed was 198.

All parents of under-five children were included in study until the required sample size of 198 was achieved. Samples were selected from medical ward of Kanti Children’s Hospital, irrespective of their ARI status. A total of 190 mothers and 8 fathers participated in the study.

### Assessment of the Variables

Exclusive breastfeeding was considered as an effect modifier. A child was considered to be exclusively breastfed if he or she was given only mother’s milk, not even water, for at least 6 months (28). Household passive smoking was the explanatory variable. Household passive smokers were those with the presence of any smoking members in their house, regardless of place where they smoked. To assess ARI, parents were asked whether their child had been ill with a cough in the 2 weeks prior to the survey. Those answering yes were additionally asked if their child, when ill with cough, breathed faster than usual with short, rapid breaths. Children who suffered from a cough accompanied by short and rapid breathing at any time during the last 2 weeks were defined as having ARI (29, 30).

### Data Collection Procedure

A semi-structured questionnaire was used to collect data. Two enumerators with a background in health were hired to collect data. Enumerators received training from the Principal Investigator. They were trained to conduct interview with emphasis on issues concerning techniques for interview (privacy, confidentiality, and reliability), taking control of the interview, and dealing with the sensitive parts of the questionnaire. They were also trained on maintaining consistency of language and terminology while interviewing.

Study respondents were interviewed face-to-face during their attendance with the doctor. The interview took approximately 15–20 min. The questionnaire included questions on (1) general characteristics of the study child (age, sex, ethnicity, area of residence, exclusive breastfeeding, and prevalence of ARI at any time in the last 2 weeks preceding the survey), (2) household situation (family size, type of house, separate kitchen, crowding, and type of fuel used for cooking and heating), (3) sociodemographic characteristics of child’s parents (education and occupation), and (4) smoking habits of family members. Most of the questions were closed with few mixed type.

### Data Analysis

Descriptive analysis was done in terms of frequency, percent, mean, and SD. Categorical variables were presented as percentage and frequency. Mean and SD were displayed in continuous variables. Effect modification of exclusive breastfeeding on association between household passive smoking and ARI was analyzed in bivariate analysis using chi-square test/Fisher’s exact test. In the final model, age, sex, and area of residence were adjusted to check the effect modification, if any, using multivariable logistic regression method. Data entered in SPSS full version 19 were analyzed in R version 3.1.2. The results were presented with adjusted OR and 95% confidence interval (CI).

### Research Ethics

Ethical approval was obtained from Institutional Review Committee of Kanti Children’s Hospital, Kathmandu, Nepal. Verbal consent was obtained from the parents of under-five children. The purpose of the study was explained to study respondents before collecting data. They were informed about their right to refuse or participate in the study and to withdraw from the study at any time. Confidentiality of information was maintained.

## Results

### Sociodemographic Features

Male to female ratio was 1.08:1 in the sample of 198 children. Mean age was computed to be 28.8 ± 11.6 months. Approximately 60% of children (male: 33.8% and female: 26.3%) were aged 2 years and above. The 40.9% children belonged to the disadvantaged janajati caste, with males and females comprising 23.2 and 17.7%, respectively. The majority was Hindu (males: 35.4% and females: 35.9%). Likewise, 46.5% male children and 44.4% female children were living in a house owned by their parents (Table 1).

**Table 1:** **Sociodemographic characteristics, household passive smoking, exclusive breastfeeding, and ARI among under-five children**.

	Male		Female		*p*-value
	
	*n*	%	*n*	%
**Age (months)**					
<24	36	18.2	43	21.7	0.139
25–59	67	33.8	52	26.3
**Ethnicity**					
Dalit	10	5.1	6	3.0	0.345
Disadvantaged Janajati	46	23.2	35	17.7
Disadvantaged non-dalit (Terai)	7	3.5	11	5.6
Religious minorities	3	1.5	5	2.5
Relatively advantaged Janajati	7	3.5	3	1.5
Upper caste	30	15.2	35	17.7
**Religion**					
Hindu	70	35.4	71	35.9	0.442
Muslim	3	1.5	5	2.5
Buddhist	17	8.6	11	5.6
Christian	13	6.6	8	4.0
**Education of mother**					
Illiterate	7	3.5	4	2.0	0.326
Up to primary	22	11.1	13	6.6
Up to secondary	47	23.7	45	22.7
Higher secondary and above	27	13.6	33	16.7
**Mother employed**					
Yes	72	36.4	60	30.3	0.314
No	31	15.7	35	17.7
**Area of residence**					
Rural	58	29.3	45	22.7	0.208
Urban	45	22.7	50	25.3
**Type of housing**					
Owned	92	46.5	88	44.4	0.418
Rented	11	5.6	7	3.5
**ARI**					
Yes	84	42.4	63	31.8	0.014
No	19	9.6	32	16.2
**Exclusive breastfeeding**					
Yes	57	28.8	56	28.3	0.608
No	46	23.2	39	19.7
**Passive smoking**					
Yes	39	19.7	40	20.2	0.543
No	64	32.3	55	27.8

*% calculated is among the total samples*.

### Prevalence of ARI, Exclusive Breastfeeding, and Household Passive Smoking

A quarter of children (25.8%) had ARI at any time in the 2 weeks preceding the survey, of which 42.4% were males and 31.8% were females. A quarter (28.8%) of male children was exclusively breastfed, with the similar proportion in female children. A considerable proportion was passive smokers (males: 19.7% and females: 20.2%), with at least one member who smoked in their house (Table 1).

### Effect Modification of Exclusive Breastfeeding on Association between Household Passive Smoking and ARI

An analysis of data did not provide any sufficient evidence on modifying effect of exclusive breastfeeding on association between household passive smoking and ARI (Tables 2 and 4). An association between the presence of anyone smoking at the house of a child (household passive smoking) and ARI was observed with adjusted odds of 4.8 (95% CI: 1.7–13.2) among non-exclusively breastfed children, while for exclusively breastfed children, this was 1.6 (95% CI: 0.6–4.3). Non-exclusively breastfed children whose family members smoked in their presence were also more likely to suffer from ARI (aOR = 6.4, 95% CI: 1.1–38.3) (Table 4).

**Table 2:** **Bivariate analysis: household passive smoking and ARI among exclusively and non-exclusively breastfed under-five children**.

Household passive smoking	ARI		ARI		MH-OR	Homogeneity test *p*-value
	Exclusively breastfed)		(Non-exclusively breastfed)			
	Crude OR	95% CI	Crude OR	95% CI		
**Presence of anyone smoking at house of child**						
Yes	1.7	0.6–4.9	4.5	1.6–13.7	2.8 (1.4–5.4)	0.165
No						
**No. of smokers in child house**						
More than one person	0.3	0.1–2.2	2.2	0.5–11.4	1.0 (0.4–2.7)	0.082
One person						
**Smoking in presence of child**						
Yes	4.6	0.5–226.7	5.1	0.9–36.9	5.1 (1.4–18.2)	0.929
No						
**Daily smoker in child house**						
Yes	0.4	0.1–2.1	1.4	0.3–6.1	0.8 (0.3–2.1)	0.23
No						
**No. of cigarettes smoked daily by family members of child**						
More than or equal to 10	0.3	0.0–6.4	0.2	0.0–1.7	0.2 (0.0–0.9)	0.763
One to nine						
**Person living in same room with child smoking in family**						
Yes	1.3	0.2–9.0	0.3	0.0–4.5	0.8 (0.2–2.8)	0.341
No						

### Indoor Pollution and ARI: An Effect Modification by Exclusive Breastfeeding

After running bivariate (Table 3) and multivariable analysis (Table 5), there was no any evidence of effect modification by exclusive breastfeeding on the association between indoor pollution and ARI. It was found that being exclusively breastfed reduced the occurrence of ARI in presence of separate kitchen in house (aOR = 0.2, 95% CI: 0.1–0.6). Children whose mothers spent at least 2 h in kitchen per day were found to experience ARI more frequently, whether exclusively breastfed (aOR = 4.5, 95% CI: 1.5–13.1) or non-breastfed (aOR = 4.5, 95% CI: 1.4–14.2) compared to children whose mothers spent <2 h in the kitchen per day (Table 5).

**Table 3:** **Bivariate analysis: indoor pollution and ARI among exclusively and non-exclusively breastfed under-five children**.

Indoor pollution	ARI		ARI		MH-OR	Homogeneity test *p*-value
	(Exclusively breastfed)		(Non-exclusively breastfed)			
**Separate kitchen in house**						
Yes	0.2	0.1–0.7	0.9	0.3–2.9	0.5 (0.2–1.0)	0.073
No						
**Place where child is usually during cooking**						
With cook in kitchen	1.3	0.5–3.6	0.8	0.3–2.3	1.0 (0.5–2.0)	0.505
Out of eye site of cook						
**Child usually carried by mother on back during cooking**						
Yes	1.5	0.5–4.5	0.3	0.1–1.2	0.8 (0.4–1.6)	0.052
No						
**Cooking in same room where child sleeps**						
Yes	1.5	0.4–5.2	0.61	0.1–2.1	0.9 (0.4–2.1)	0.273
No						
**Fuel used during cooking**						
Unclean fuel	1.2	0.5–3.0	0.5	0.2–1.4	0.8 (0.4–1.5)	0.536
Clean fuel						
**Fuel used for heating**						
Unclean fuel	0.8	0.3–1.9	1.3	0.5–3.2	1.0 (0.5–1.8)	0.945
Clean fuel						
**Time spent by mother in kitchen per day**						
2 h and above	3.9	1.8–10.6	4.4	1.6–12.1	–	0.000
Less than 2 h						

## Discussion

The current study indicated that non-exclusive breastfeeding increases the effect of household passive smoking on ARI. As a result, integrating an exclusive breastfeeding component on child health programs could be beneficial. Nevertheless, on account of wider CIs in adjusted estimates, cautious interpretation is needed prior to generalization of study findings.

The presence of anyone smoking in the house of child increased the possibility of ARI among non-exclusively breastfed children. The condition was similar for non-exclusively breastfed children whose family members smoked in their presence (Table 4). A previous study by Woodward and colleagues reported seven times higher odds of ARI among never breastfed children who were living with smoking mothers compared to breastfed ones (19).

**Table 4:** **Household passive smoking and ARI among exclusively and non-exclusively breastfed under-five children**.

Household passive smoking	ARI		ARI	
	(Exclusively breastfed)		(Non-exclusively breastfed)	
	Adjusted OR	95% CI	Adjusted OR	95% CI
**Presence of anyone smoking at house of child**				
Yes	1.6	0.6–4.3	4.8	1.7–13.2
No				
**No. of smokers in child house**				
More than one person	2.9	0.5–17.8	0.4	0.1–1.9
One person				
**Smoking in presence of child**				
Yes	5.1	0.5–56.6	6.4	1.1–38.2
No				
**Daily smoker in child house**				
Yes	0.6	0.1–3.0	1.4	0.4–5.6
No				
**No. of cigarettes smoked daily by family members of child**				
More than or equal to 10	–	–	1.5	0.1–20.8
One to nine				
**Person living in same room with child smoking in family**				
Yes	1.1	0.2–6.4	0.2	0.0–3.0
No				

*Adjusted for age, sex, and area of residence*.

The finding of a higher risk of ARI among non-exclusively breastfed children could be due to lowered immunity among these children, given that breast milk has been shown to protect against infection (31–33). It is also possible that among exclusively breastfed children, there is dilution of the effect of passive smoking on ARI due to long-lasting immunity provided by exclusive breastfeeding, while non-exclusively breastfed children lack this advantage. Breast milk tends to have different immunomodulatory factors which, in addition to their other biological and antimicrobial functions, are able to actively regulate the maturation of the immune system of the neonate (34). Higher risk of ARI among non-exclusively breastfed children could also be because of being deprived of broad-based beneficial effect of exclusive breastfeeding in prevention of infectious diseases, such as diarrhea, that often contribute to development of malnutrition, further leading to immunologic insufficiency (16).

On aggregate sample, children with separate kitchen in house had lower chances of ARI as shown by the original study (23), which also corresponds to previous studies (35). However, when disaggregating by breastfeeding status, having a separate kitchen in house had protective effect against ARI only among the exclusively breastfed children (Table 5). It is possible that when there is separate kitchen in household, there is reduced indoor air pollution, thereby reducing the chance and duration of exposure to pollutants reducing the incidence of ARI. These effects are more pronounced among exclusively breastfeed children. However, it must be noted that in this study, children with a separate kitchen in their house also had lower rates of exposure to other enabling factors for ARI compared to those without separate kitchen: rural residents (48.3 vs. 63.8%), use of unclean fuel for cooking (54.3 vs. 72.3%), cooking in the same room where child sleeps (1.3 vs.78.7%), and carrying baby on back while cooking (23.2 vs. 40.4%) (table not shown). These might have also played a role separately or collectively in the development of ARI.

**Table 5:** **Indoor pollution and ARI among exclusively and non-exclusively breastfed under-five children**.

Indoor pollution	ARI		ARI	
	(Exclusively breastfed)		(Non-exclusively breastfed)	
	Adjusted OR		Adjusted OR	
**Separate kitchen in house**				
Yes	0.2	0.1–0.6	0.7	0.2–2.2
No				
**Place where child is usually during cooking**				
With cook in kitchen	0.9	0.3–2.6	0.8	0.3–2.3
Out of eye site of cook				
**Child usually carried by mother on back during cooking**				
Yes	1.3	0.4–3.7	0.3	0.1–1.0
No				
**Cooking in same room where child sleeps**				
Yes	1.9	0.5–6.3	0.7	0.2–2.4
No				
**Fuel used during cooking**				
Unclean fuel	2.6	0.6–10.1	0.4	0.1–1.7
Clean fuel				
**Fuel used for heating**				
Unclean fuel	0.4	0.1–1.5	1.2	0.3–4.1
Clean fuel				
**Time spent by mother in kitchen per day**				
2 h and above	4.5	1.5–13.1	4.5	1.4–14.2
Less than 2 h				

*Adjusted for age, sex, and area of residence*.

Time spent by mother in kitchen, for at least 2 h per day, played a crucial role in ARI outcome, regardless of whether the child was exclusively or non-exclusively breastfed (Table 5). It is logical to assume that longer the time spent in kitchen by mother, the longer her child is exposed to indoor air pollution, thereby increasing the risk of ARI through a dose–response relationship. This could be significant, with the fact that just 27.3% mothers reporting regular carrying of babies on back while cooking food in the present study. Most importantly, the majority of the babies (around 60%) carried were <2 years of age (table not shown). Studies suggest that infants (36) and children <2 years of age (35) are more prone to ARI. In addition, the majority of parents who reported time spent by mother in kitchen for at least 2 h per day used unclean fuels for cooking (67.7%), which is one of the risk factors of ARI (37). This calls for health education programs on deleterious effects of mother’s longer exposure to cooking smoke on child’s health.

Before interpreting the findings of the current study, various other things need to be considered. Information on ARI was solely based on parent’s reporting about the ARI status of children and no clinical measurements were undertaken. Clinical data on ARI are usually not available in Nepal; therefore, ARI was assessed based on symptomatic definition. This definition has been used in many other previous studies (29, 30). There were potential limitations in this study: small sample size, hospital-based study, lack of validation regarding passive smoking exposure, and no validation of ARI by doctor. As this was a hospital-based study, the sampling proportion of exclusively and non-exclusively breastfed children may not be the true proportion of exclusively and non-exclusively breastfed children in the entire population.

Sample size was initially calculated with the aim of measuring association, not accounting for interaction effect by exclusive breastfeeding. Therefore, it was likely that study was underpowered to detect this interaction effect. Also, we did not consider for multiplicity testing.

Likewise, the cross-sectional design may not provide sufficient evidence to draw an inference on causal association. Further, results of the current study was based on adjustment of limited factors (sex, area of residence, and age), questioning the chances of confounding by other known and unknown potential factors, as this was a small sample study with restricted ability to account for confounding factors.

Even so, with the evidence of substantial under-five household passive smoking children (26), and not universal exclusive breastfeeding practice (22), having an insight into effect of household passive smoking on ARI among exclusively and non-exclusively breastfed children could aid in promoting cost-effective interventions, such as exclusive breastfeeding, in Nepal. Large prospective studies with a larger sample size are needed to confirm effect modification by exclusive breastfeeding on the association between household passive smoking and ARI.

## Conclusion

This study found that the lack of exclusive breastfeeding may enhance the effect of household passive smoking on ARI in children under-five. In both exclusively and non-exclusively breastfed children, the time spent by mother in the kitchen played a vital role in ARI outcome. However, the modifying effect of exclusive breastfeeding on the association between household passive smoking and ARI was not detected. Further research with larger sample size may be useful for testing hypothesis on whether exclusive breastfeeding modifies the effect of household passive smoking on ARI among under-five children.

## Author Contributions

PT conceptualized the study, acquired the data, analyzed data, and wrote the first draft of the manuscript. AP, RD, BB, BT, and SM carried out data analysis, interpretation of findings, and reviewed the manuscript. All authors gave their approval to the final manuscript.

## Conflict of Interest Statement

The authors declare that the research was conducted in the absence of any commercial or financial relationships that could be construed as a potential conflict of interest. The reviewer, TKJ, and handling Editor declared their shared affiliation, and the handling Editor states that the process nevertheless met the standards of a fair and objective review.
